# Evaluating Letter Recognition, Flicker Fusion, and the Talbot-Plateau Law using Microsecond-Duration Flashes

**DOI:** 10.1371/journal.pone.0123458

**Published:** 2015-04-13

**Authors:** Ernest Greene

**Affiliations:** Laboratory for Neurometric Research, Department of Psychology, University of Southern California, Los Angeles, California, United States of America; New York University, UNITED STATES

## Abstract

Four experiments examined the ability of respondents to identify letters that were displayed on an LED array with flashes lasting little more than a microsecond. The first experiment displayed each letter with a single, simultaneous flash of all the dots forming the letter and established the relation of flash intensity to the probability of letter identification. The second experiment displayed the letters with multiple flashes at different frequencies to determine the probability that the sequence of flashes would be perceived as fused. The third experiment displayed the letters at a frequency that was above the flicker-fusion frequency, varying flash intensity to establish the amount needed to elicit a given probability of letter identification. The fourth experiment displayed each letter twice, once at a frequency where no flicker was perceived and also with steady light emission. The intensity of each flash was fixed and the steady intensity was varied; respondents were asked to judge whether the fused-flicker display and the steady display appeared to be the same brightness. Steady intensity was about double the average flash intensity where the two conditions were perceived as being equal in brightness. This is at odds with Talbot-Plateau law, which predicts that these two values should be equal. The law was formulated relative to a flash lasting half of each period, so it is surprising that it comes this close to being correct where the flash occupies only a millionth of the total period.

## Introduction

The Talbot-Plateau law [1–4] asserts that a flash sequence that is above the flicker-fusion threshold will be perceived as being equal in brightness to a steady stimulus when the average intensity of the former matches the intensity of the latter. The average across both light and dark periods of the flash sequence specifies the quantity of photons being delivered across each light/dark cycle. Most of the support for this principle has used a 50/50 ratio of light and dark, as detailed in Discusson, and it would be useful to gather additional data in the microsecond range. The present work required respondents to identify letters of the alphabet that were displayed with brief flashes, and examined flicker fusion and the Talbot-Plateau law under these stimulus conditions.

Previous work from this laboratory asked for recognition of natural and constructed objects, *e*.*g*., animals, vehicles, tools, and furniture, which were represented by an array of dots that marked the outer boundary of each object. [5] The shapes could be identified when displayed using a single, simultaneous flash of all the dots, even with flash durations as brief as three microseconds. The present experiments displayed discretized letters rather than outline boundaries of complex shapes, and used flashes with a duration of 1.3 microseconds (μs). The first experiment established the light intensity required to elicit various levels of letter recognition as a function of intensity.

The goal of the second experiment was to determine at what frequency a sequence of brief flashes will fuse into the perception of a steady, non-flickering stimulus, hereafter described as a fused-flicker display. This experiment derived a plot of flicker detection as a function of flash frequency using an intensity that was effective at eliciting letter recognition in Experiment 1.

Experiment 3 assessed the relative salience, *i*.*e*., ability to elicit recognition, of 24 Hz flicker displays—these being above the flicker-fusion threshold that was manifested in Experiment 2. By delivering many more flashes over an extended period, a fused-flicker display is expected to be far more effective at eliciting recognition than a single flash at the same intensity. Whereas a single flash at threshold intensity produces minimal recognition of objects, two successive flashes at the same intensity can elicit near maximal recognition [6]. A stream of flashes that is perceived as a steady display should be stronger yet. Experiment 3 quantified the salience of a 24-Hz fused-flicker sequence of brief flashes, establishing the intensities required to produce a given probability of recognition from threshold through maximal levels.

The final task (Experiment 4) compared 24-Hz fused-flicker displays with steady displays, using a fixed intensity for each flash in the sequence and varying the intensity of the steady display. In addition to identifying the letters that were shown, respondents judged whether the two were comparable in brightness.

## Results and Discussion

### LED Display of Letters

**Fig 1:**
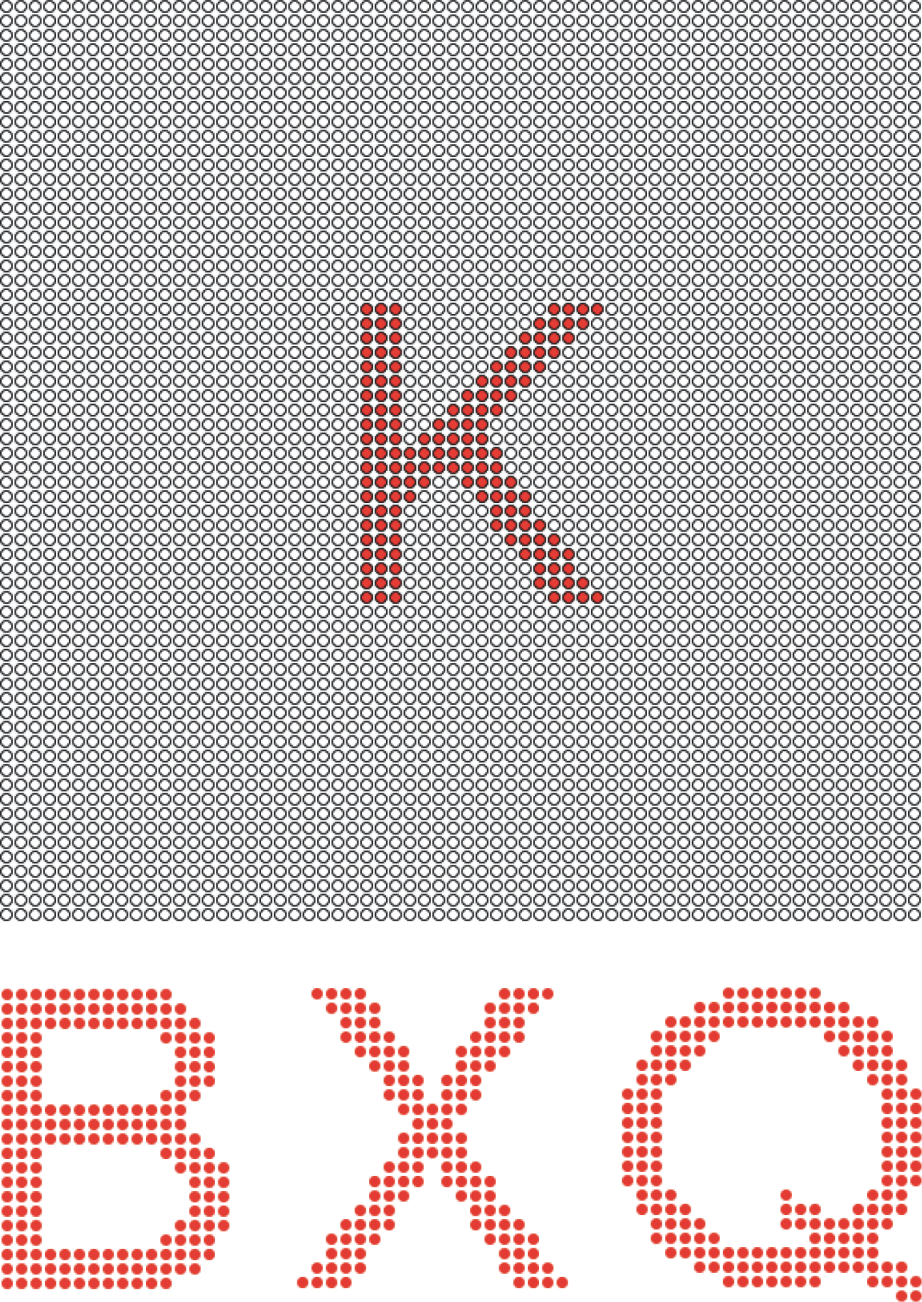
Discretized versions of letters were displayed on an array of LEDs with brief single or multiple flashes or with steady emission of light. Four examples are provided here; the letter K shows how a letter would be positioned within the array.

In each of the experiments that follow, letters from the standard English alphabet were displayed using an array of light-emitting diodes (LEDs), as illustrated in Fig 1. A given letter could be displayed with a single and simultaneous brief flash of light from each of the LEDs forming the letter pattern, as a sequence of brief flashes, or as steady display of the pattern for a specified period of time. In each of the experiments, respondents were required to name the letter that was displayed. In Experiments 2 and 4 they provided additional judgments about perceived flicker and brightness, respectively. There were eight respondents in each of the four experiments—a total of 32 respondents. Light intensity is reported in radiometric units of intensity. If one wants conversion to photometric units, the μW/sr values can be divided by 5.6 to get mCd values. Earlier work had taken measures of pupil diameter under identical ambient and display conditions, finding a mean diameter of 6.6 mm. This allows one to calculate Trolands by dividing the radiometric value by 1922.

For each of the experiments, judgment probabilities were modeled using random effects semi-parametric logistic regressions (see Methods for details). Each group model was found to be a significant departure from chance at p < 0.0001.

### Recognition of Letters as a Function of Flash Intensity

The first experiment derived activation curves for each of the respondents as a function of flash intensity, as well as a mean model for the group. The experiment sampled intensities across a specific range, which would be classified as a “constant stimulus” psychophysics protocol. Treatment levels were the same for all respondents. Each of the 26 letters was displayed at each treatment level, with both the order of treatments and the order of letters within a given treatment being chosen at random. With each display the respondent was required to name the letter that had been shown and was not provided with any feedback as to the validity of the response. Across treatment levels the probability of correct identification, designated here as hit rate, is essentially a frequency-of-seeing curve that is deeply rooted in classic psychophysics.

**Fig 2:**
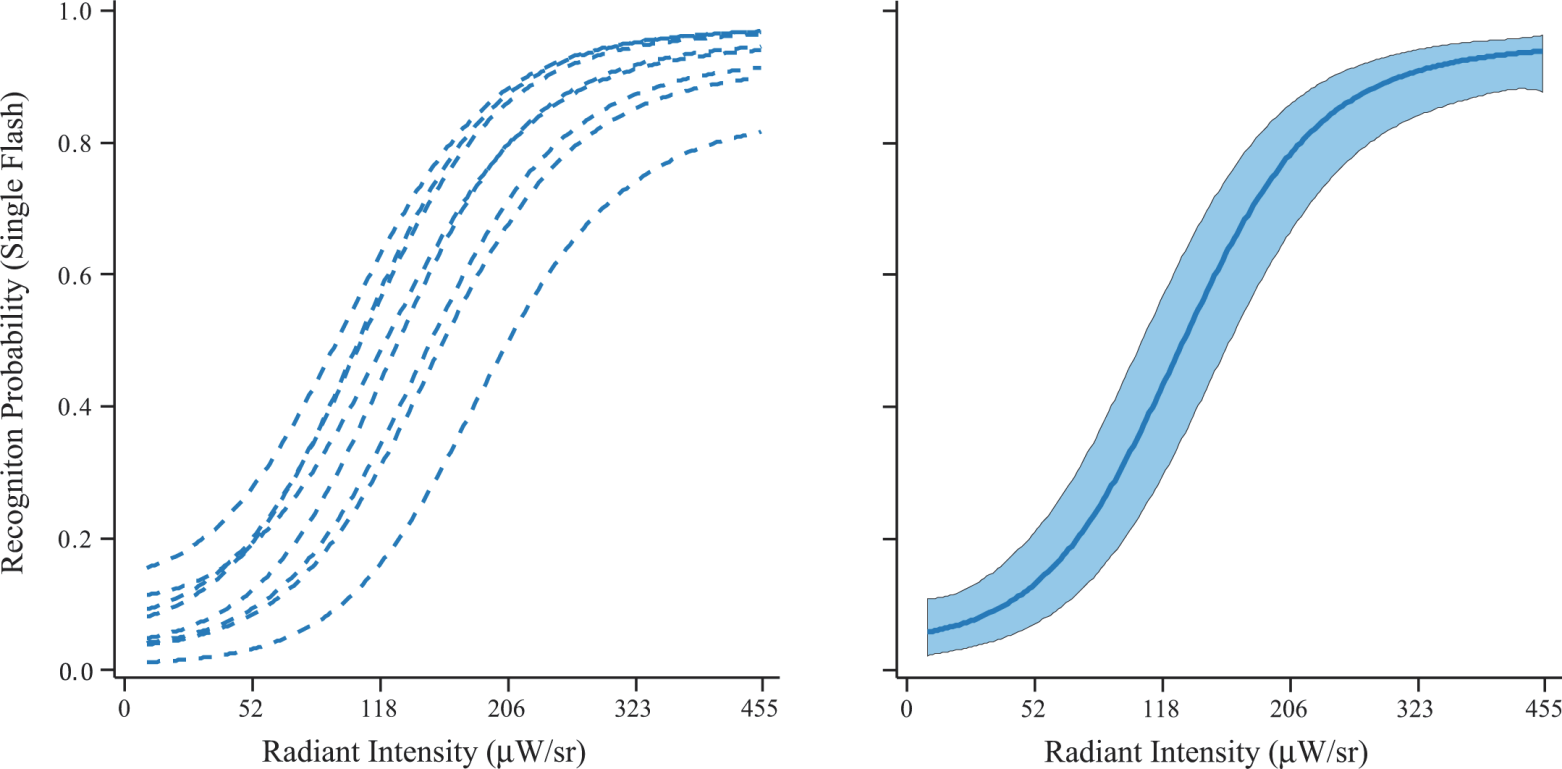
In Experiment 1 respondents were asked to identify letters that were displayed with a single flash with intensity being varied. Statistical models showing the probability of recognition against flash intensities are plotted in the two panels. Individual models for the eight respondents are shown on the left. Each showed a monotonic rise in recognition as intensity of the flash was increased. The right panel shows the group model along with a 95% confidence band.

Models for each of the eight respondents tested in Experiment 1 as well as the overall model for the group are shown in Fig 2. Each of the respondents manifested a monotonic rise in probability of letter recognition, with hit rates that were in the chance range at low intensities and were at or very near 100% at the top end.

### Flicker Fusion with Multiple Flashes

Experiment 2 tested respondents for recognition of letters that were displayed using a sequence of flashes. Each brief flash in the sequence displayed all the dots of a given letter, as in Experiment 1. Flash intensity was set at the mean level at which the respondents in Experiment 1 first reached their maximum hit rate. The frequency of these flashes was varied from 6 Hz to 30 Hz, with the entire flash sequence being continued for 750 milliseconds.

As one of the measures taken in each experiment, respondents were required to identify each letter, this being simply to affirm a suitable focus of attention to the tasks. Given that a quantity of photons near the high end of the range in Experiment 1 elicited near perfect recognition, the expectation for Experiment 2 was that the multiple sequence would provide the same high level of performance. This was confirmed—across all frequency conditions the mean hit rate was 0.975.

**Fig 3:**
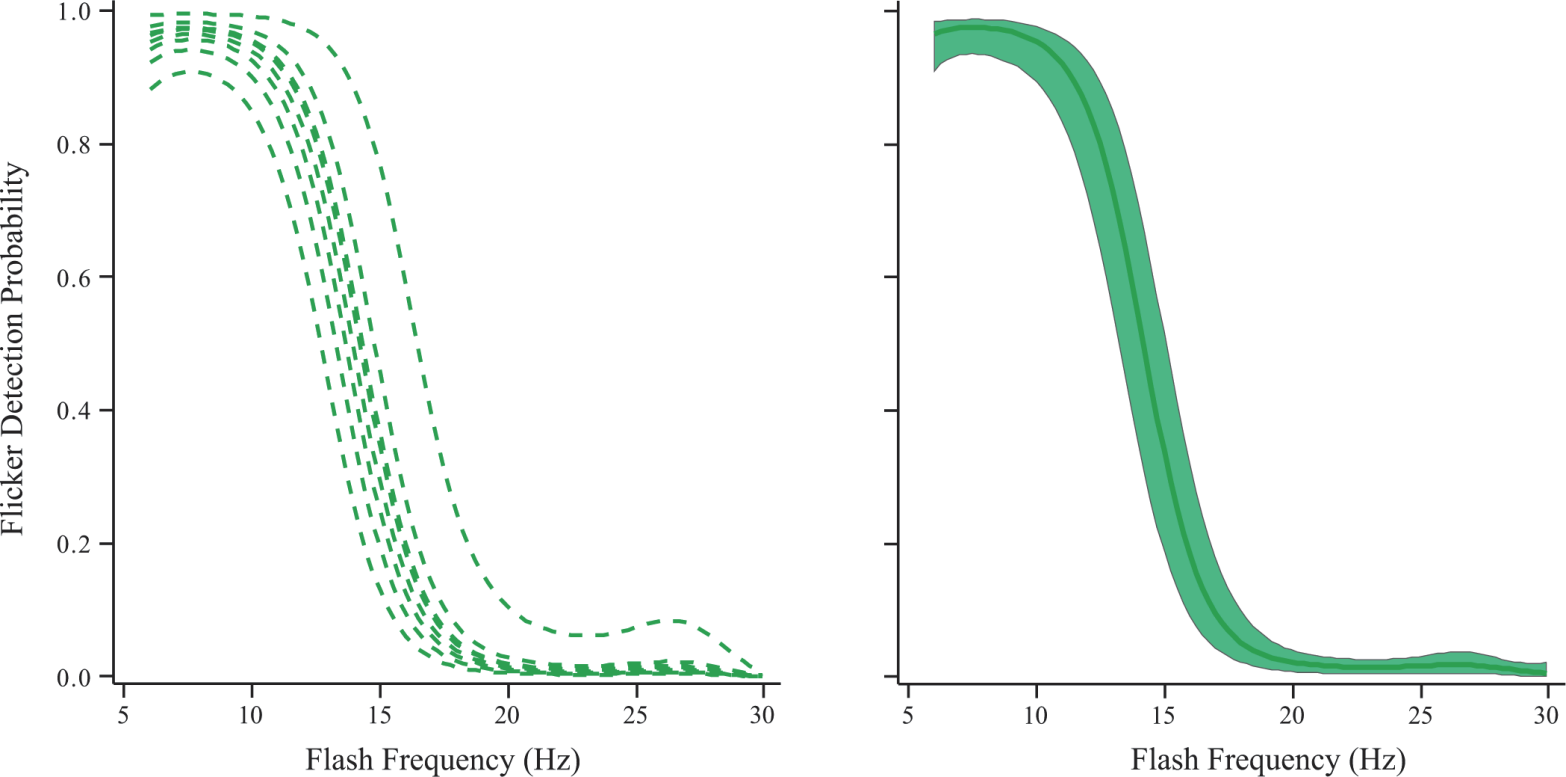
In Experiment 2 letters were displayed with multiple flashes across a duration of 750 ms, varying flash frequency. Letters could be reliable identified irrespective of frequency, but the key question here was at what frequencies would the flashes be seen as fused, *i*.*e*., appearing as steady emission. The probability of flicker detection is given on the ordinate against flash frequency on the abscissa of each panel. The individual models (left panel) shows that respondents saw the sequence of flashes as flickering at frequencies below 10 Hz and the flashes appeared as steady non-flickering emission at frequencies above 20 Hz. The right panel shows the group model and its 95% confidence band.

The judgment of real interest for Experiment 2 was an assessment of whether the flash sequence was perceived as having flicker or as providing steady emission. The frequency range—6 to 30 Hz—had been chosen with the expectation that respondents would reliably see flicker at the low end of the frequency range and not see any flicker at the high end. This was confirmed, as can be seen in Fig 3. The individual respondent and mean models all show consistent sigmoid functions across the range, with a sharp decline in the probability of flicker detection that begins at about 10 Hz and is at or near zero by about 20 Hz. By convention the critical flicker frequency is that which produces flicker detection 50% of the time, which was at 14 Hz for the present data.

It is well established that plots of flicker-fusion thresholds have two limbs, reflecting differential activation of rods and cones [7]. Rods generate long-duration persistence in response to low intensity flashes, which in turn provides for fusion at a low flicker frequency. Cones respond at higher light intensities, generating shorter-duration persistence and manifesting fusion at higher frequencies. The present display system used LEDs that emit near the end of the visible spectrum, with peak emission at 630 nm and with the range of wavelengths being very narrow. This stimulus would activate red cones. would have a limited influence on green cones, and would not likely influence blue cones or rods. [8,9]

The flicker-fusion threshold was 14 Hz, very low on the cone-response curves reported by various investigators [7,10–16]. This would be expected if flash intensities were in the scotopic range, but here the ambient illumination was mesopic and the intensity was high enough to allow reliable recognition of the letters. It is possible that the low fusion frequency was due to the unusually low duty cycle, *i*.*e*., a very short flash duration relative to the full period from one flash to the next, as discussed below.

### Recognition as a Function of Intensity with Fused-flicker Flashes

The goal of Experiment 3 was to establish the range of intensities that provide for low to high levels of recognition with fused-flicker displays. Each letter was displayed using a 24 Hz sequence of brief flashes for a total duration of 750 milliseconds. This frequency was well above the level that assured fusion for each of the respondents in Experiment 2, and on being questioned, each of the respondents in Experiment 3 said the light emission appeared to be steady.

**Fig 4:**
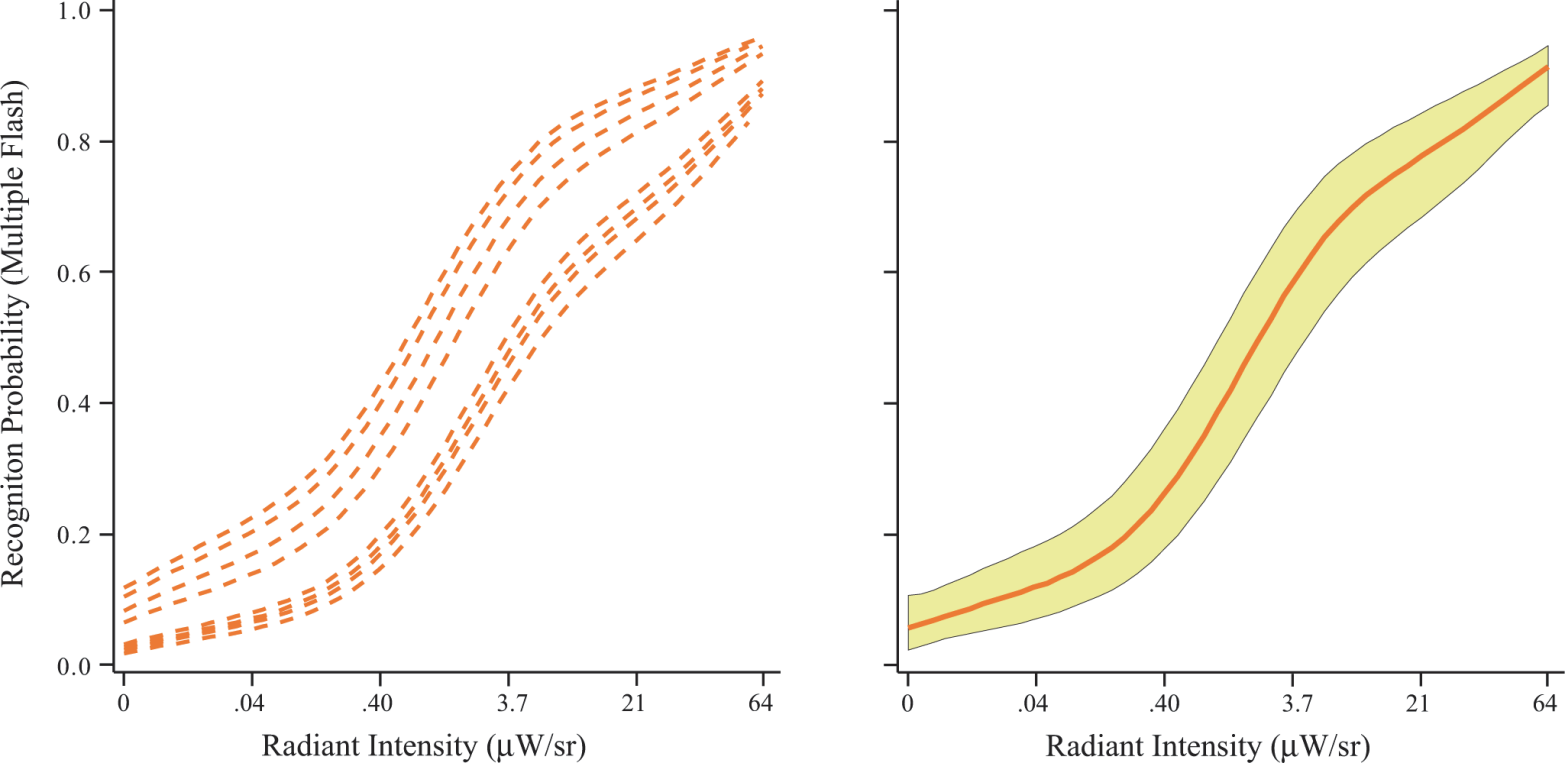
In Experiment 3 each letter was displayed with a perceptually fused sequence of flashes at 24 Hz for 750 ms, and intensity was varied to determine how the probability of recognition changed. The left panel shows the individual models of respondents; each showed a monotonic rise in hit rate with increasing flash intensity. The right panel shows the group model plus the 95% confidence interval.

Fig 4 shows the modeling results, with probability of recognition being plotted against flash intensity. As in Experiment 1, each of the respondents manifested a monotonic rise in hit rate as intensity was increased. As expected, the intensities needed to produce comparable levels of recognition were much lower than for Experiment 1. At the low end of the activation function the difference is about 7 to 1 and at the top end the ratio is about 5 to 1. In the first experiment the respondents identified letters on the basis of a single flash of all the dots in the letter-pattern. In Experiment 3 the letter was flashed 18 times (24 Hz for 750 ms). Prior work from this laboratory has shown that an intensity that is minimally effective at eliciting recognition when delivered by a single flash can produce very high levels of recognition when two flashes are provided. [6] Therefore it is not surprising to find that a sequence of 18 flashes require much less intensity to produce comparable levels of recognition.

### Flash Activation and Fusion

Neurons are able to generate impulses at far higher frequencies than the frequencies at which flicker-fusion is observed, so the limitation on temporal resolving power is usually attributed to some form of persistent neuronal activity. The perceptual manifestation can be described as visible persistence, and “sustained” firing of neurons is generally assumed to be the mediator of that persistence. There has been an ongoing debate as to whether the fusion takes place in the retina or at a later processing stage. Walker and associates [17] registered neuronal responses with needle electrodes, recording from optic nerve, lateral geniculate nucleus, tectum, optic radiations, and striate cortex of macaques. Individual flashes could be followed in the optic nerve and lateral geniculate nucleus up to about 60 Hz but the cortex never followed photic driving above 34 Hz. This is about the frequency at which Brecher [18] found flicker fusion in the monkey, so Walker and associates [17] thought it likely that fusion takes place in cortex.

However, Pinneo & Heath [4] provide evidence that differential firing rates of ganglion cells may determine the frequency at which fusion occurs. They recorded from two patients with depth electrodes that had been implanted for therapeutic purposes. Electrodes likely recorded electrical activity from the optic tract in the first patient and from the lateral geniculate nucleus in the second patient. Neither patient had visual impairments. They were stimulated with steady light at 2.5 lux and with a flicker stimulus with a 50% duty cycle that had a mean intensity of 2.5 lux. Flicker frequency ranged from one to 50 Hz and the patients reported whether or not they saw flicker. The level of spontaneous neuronal activity in response to steady illumination was at the same level irrespective of the level of dark adaptation, *i*.*e*., it was a function of the intensity of the steady light and not the level of dark spontaneous activity just preceding light onset. With onset of a perceived flicker, net activity measured from the optic tract of the first patient rose with each increase in frequency up to a peak at 12 Hz. Above 12 Hz the frequency of firing decreased with each step up to about 33 Hz, after which the activity level became constant for all higher frequencies. The level of activity at 33 Hz and beyond was the same as for a 2.5 Hz steady light. The subjective perception of the stimulus as being “steady” took place at around 32.5 Hz, and it remained so at all higher frequencies. The second patient provided comparable results. Overall, their findings seem to argue for differential of firing rate in optic nerve fibers providing the basis for perceived fusion of a flickering stimulus.

Under dark adapted conditions, a 20 Hz flicker fusion threshold is at the transition between rod and cone threshold functions. At the lowest flash intensities the rods are providing for perception of the flicker, and the flashes fuse at frequencies well below 20 Hz [7,11–13]. At higher flash intensities the response of rods asymptotes near 20 Hz, after which the cones are providing the flicker judgment. Using color-filtered test fields Hecht & Shlaer [7] found the rods to be sensitive to wavelength, but cone thresholds were remarkably unresponsive to wavelength differentials.

It is undoubtedly relevant that the present work used exceptionally short flash durations, and there are indications that duration is a factor in the persistence of flash influence. Using white-light flashes and varying flash duration across a range from 10% to 90%, *i*.*e*., from 100 ms to 900 ms per one-second interval, Crozier & Wolf [12] found the flash intensity required to produce fusion was increased as the percentage of light in the duty cycle was increased. This means that persistence of flash influence is longer with shorter-duration flashes. They describe the results as an “increase in the efficiency of briefer flashes.” Crozier & Wolf [13] examined 10% versus 90% duty cycles as a function of wavelength. Across all conditions the flicker-fusion curves were comparable in shape, but the shorter-duration flashes fused at lower flash intensities for each wavelength that was tested. At a given intensity the shortest flashes were the most effective at eliciting visible persistence, which allowed the flashes to be seen as steady illumination at a lower frequency.

The findings of Battersby & Jaffe [19] support the concept that duty cycle (light/dark ratio) is a major determinate of flicker fusion frequency [see also 14,15,20,21]. Battersby & Jaffe [19] examined for differentials of critical flicker frequency with light/dark ratios of 20, 50, and 80% and found that the slopes of the flicker-fusion plots varied as a linear function of “light energy.” For each light/dark ratio an increase in the total energy of the flashes produced an increase in the frequency at which fusion occurred. However, the energy required to produce each plot was inversely related to the size of the light/dark ratio. The flickers having the smallest light/dark ratios generated the full plot of flicker-fusion thresholds on the basis of lower energy range than was required for flashes with higher light/dark ratios.

### Comparability of Brightness with Steady and Fused-flicker Flashes

The final experiment displayed each letter twice, first as a 24 Hz fused-flicker sequence followed by a steady emission of the letter, or in the reverse order. Each display was for 750 ms with a 250 ms gap between. As in each of the prior experiments the respondents were asked to identify the letters. For this experiment the recognition hit rates ranged from 0.970 to 0.997, the mean hit rate being 0.989.

**Fig 5:**
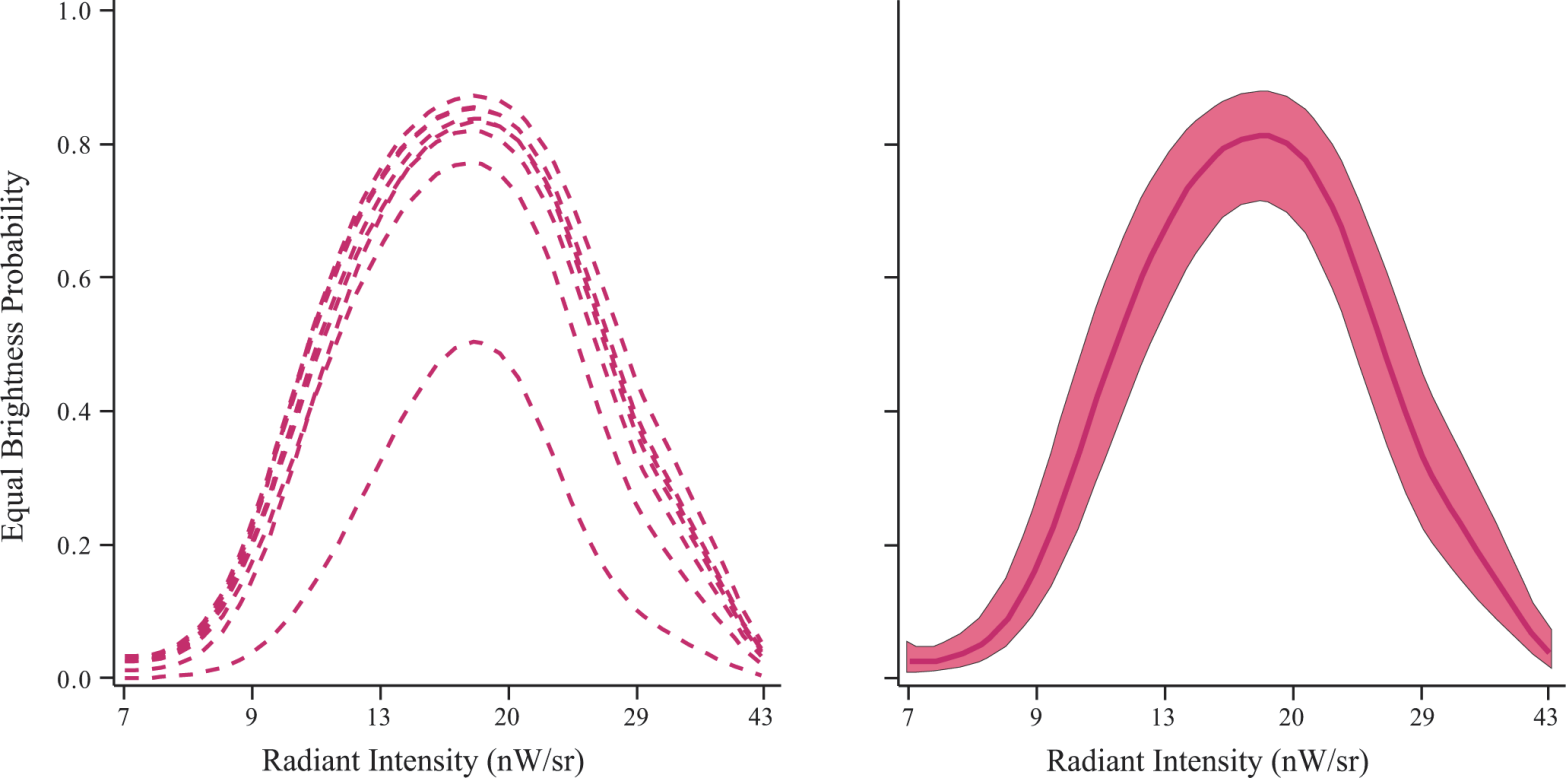
On each trial of Experiment 4 a letter was shown twice, once as a 24 Hz flash sequence and also as a steady display, each for 750 ms. The intensity of each flash was fixed, and the intensity used for steady displays was varied. [Note that scale values are in nanowatts/sr rather than microwatts/sr.] Respondents judged the two display conditions to be different in brightness at each end of the range and as the same brightness in the middle of the range. The Ferry-Porter law specifies that the average intensity of the flashes should equal the intensity of steady displays. They were not quite the same in this experiment, but the degree of match is impressive given the extreme brevity of flash durations.

The respondents also judged the relative brightness of the two display conditions, this being the main goal of the experiment. To provide conditions that allow for comparison of brightness, Experiment 4 used a fixed intensity for each flash in the fused-flicker displays and varied intensity for the steady displays. Intensity for flashes was set at the level used in Experiment 2. Then on the basis of pilot data, a range of intensities was chosen for the steady displays that would provide subjective levels of brightness that extended above and below the perceived brightness of the fused-flicker sequence. Thus on each trial a letter was displayed with a sequence of fixed-intensity flashes and also a steady display at one of the intensity levels (order of these two displays being chosen at random). Fig 5 provides models where judgments of brightness comparability (1 = same, 0 = different) is specified as a function of the steady intensities.

The expectation, confirmed by the models shown in Fig 5, was that two displays would be judged as “different” at both ends of the steady-emission range, with the plots manifesting a central peak where the displays were seen as being the “same brightness.” One respondent deviated substantially from the other seven, with a peak response where only about half of the fused-flicker displays were judged as having the same brightness as steady displays. Nonetheless, the overall shape of the model is essentially the same as for other respondents, and the model peaked at almost exactly at the same intensity as the other seven respondents. The difference in overall height of the model does not preclude the following inference.

These results support the proposition that a flash sequence that is above the flicker-fusion frequency can elicit a visual signal that is perceived as equivalent in brightness to that provided by steady stimulation of the retina. Either display provides a highly visible stimulus that allows letters to be identified.

### Intensities for Equivalent Brightness Judgments

The radiant intensities judged as being equivalent in brightness for steady and fused-flicker conditions were 1.78 x 10^−2^ and 2.88 x 10^2^ μW/sr, respectively. The two intensities differ by roughly four orders of magnitude. As noted at the outset, Talbot [1] and Plateau [2] provided evidence that these two display conditions will be perceived as equal in brightness when the intensity of the steady stimulus is equal to the average for the intermittent-flash condition, *i*.*e*., including the dark portion of the cycle in the average. In the present work each flash had a duration of 1.3 microseconds. Eighteen flashes were presented during the 750 ms interval, providing emission for less than 24 microseconds and dark for the remainder of the interval. Calculating the mean across the light and dark portions provides an average intensity of 0.90 x 10^−2^; the steady intensity is about twice this value. Therefore the present results do not fully conform to the Talbot-Plateau law.

It should be noted that flicker sequences are most often tested with a 50% duty cycle, *i*.*e*., an equal interval of light and dark within each cycle. The Talbot [1] and Plateau [2] studies used this protocol, and it is possible that the principle that they espoused is valid only for a 50% duty cycle. Nelson & Bartley [22] provide evidence that the Talbot-Plateau predictions do not hold up when the flash-train is short. They suggest that the display period likely needs to be about 2 seconds before the brightness judgments are equal. The present use of 0.75 sec displays may have contributed to deviation from the Talbot-Plateau law.

Alternatively, or in addition, the Broca-Sulzer effect may be at work [23, 24–29]. It manifests as an increment of brightness above what would be expected on the basis of flash intensity. The Broca-Sulzer effect has been described as an overshoot at the top of the rising phase, peaking with flash frequencies of about 50 milliseconds for high-luminance stimuli and in the 300 millisecond range at the low end of the intensity range [30–34]. In other words, it has been characterized as an enhancement of perceived brightness for flash durations ranging from about 50 to 300 milliseconds.

**Fig 6:**
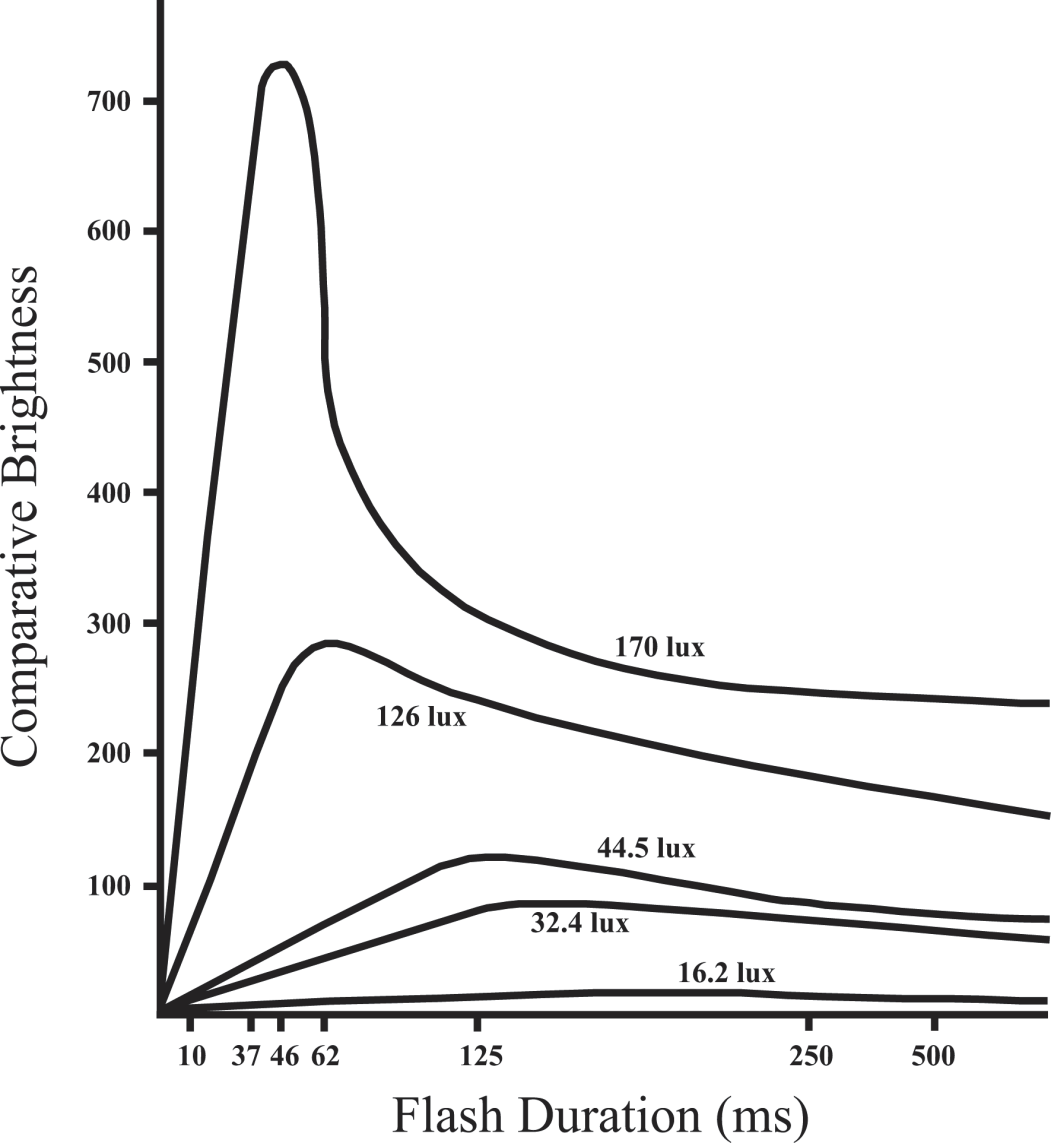
This is an adaptation of a figure provided by Broca & Sulzer [22], wherein the perceived brightness of brief flashes was compared to steady levels of light. The two were seen as approximately equal in brightness at very long intervals where the flash judgments were becoming asymptotic. At short flash durations there was a very large difference in the perceived brightness at high flash intensities. Additionally, there was a shift in where the peak was manifested, being seen at progressively shorter flash durations as flash intensity was increased.

However, the original Broca & Sulzer [23] plots provide a much clearer picture of how the brightness peaks at progressively shorter flash durations as intensity is increased (see Fig 6). Each increase in flash intensity moved the peak at which the Broca-Sulzer effect was manifested; 44.5 lux produced a peak of brightness perception with 125-millisecond flashes and 170 lux produced the peak with 46-millisecond flashes. The amplitude of peak brightness at 170 lux was over seven times larger than for 44.5 lux flashes.

The physiological mechanism responsible for these effects may be registering an interaction of flash duration and intensity. The common description that the Broca-Sulzer effect is seen in the 50–300 ms range implies that the effect is restricted to that range, yet there is no evidence that this is the case. It seems plausible that with flash durations in the microsecond range could activate Broca-Sulzer enhancement of perceived brightness. This could make the letter patterns especially salient even when the intensity being delivered is less than for steady display of the letters.

Experiment 4 respondents did judge the 24 Hz flash sequence to be equal in brightness to the steady display when the average intensity of the flash sequence was only half that of the steady display. This is not as predicted by the Talbot-Plateau law. However, it is somewhat surprising that this principle would come this close to specifying the relative effectiveness of these two conditions where each flash in the sequence lasted little more than a millionth of a second.

It is reasonable to assume that any enhancement of stimulus salience should be attributed to retinal mechanisms. One might ask whether the response of the photoreceptors differs as a function of flash duration. It is generally accepted that the rise in photocurrent is directly proportional to the total amount of photopigment that has been isomerized [35,36]. An isomerization leads to activation of cyclic GMP [37,38], which in turn migrates to the outer segment membrane, closing sodium and calcium channels and producing hyperpolarization of the photoreceptor. Lamb & Pugh [39] provide a comprehensive model of the relation of flash energy on photocurrent amplitude and duration and Korenbrot [40] provides a review of phototransduction mechanisms.

Cobbs & Pugh [41] report that flashes with durations of 22 ms produced prolonged “impulse responses” in the photoreceptors; the amplitude and duration of a given response was reported to be a function of the total number of bleached photopigment molecules. In salamander rods the photocurrent impulse response generated by a dim flash peaked in about 750 ms and returned to baseline in about 3 seconds. Photocurrents generate photovoltages, which rise to reach a peak a bit faster.

The impulse response has a faster time course in monkey cones. With a 10 ms dim flash, photovoltage reaches a peak in 32–35 ms and the hyperpolarization returns to baseline in roughly 100 ms [42,43]. Human cones reach a peak in 20 ms [44]. Rod responses are slower [42,45].

Cobb & Pugh [41] also stimulated with bright flashes lasting only 20 μs. The most intense flashes produced impulse responses that had a latency of 7.5 ms and peaked in about 40 ms, considerably faster than with 22 ms flashes. This suggests the possibility that temporal response of photoreceptors is influenced by the duration of the flash. On the other hand, Nikonov and associates [46] recorded impulse responses in mouse cones using 20 μs and 7 ms flashes and the times to peak and durations were fairly comparable.

## Methods

### Experiment Approval and Informed Consent

The experimental protocols were approved by the Institutional Review Board at USC. Each respondent was provided with a description of the nature of the research and the judgments they would be asked to make. Further, each was informed that he or she could discontinue participation at any time and for any reason (or no reason) without penalty. Respondents were free to chose among a broad range of experiments and a record of their choice has been retained by the Psychology Department, providing documentation of informed consent. Thirty-two respondents contributed data, eight in each of the four experiments.

### Letter Attributes

Arial 33-point TrueType fonts were rendered, without antialiasing, as discrete dots that were properly positioned for display on the LED array (see Fig 1). The heights and widths of letters along with the total number of dots in each letter are specified in Table 1.

**Table 1:** Letter Attributes.

Letter	width/dots	width/arc°	height/dots	height/arc°	# dots
A	19	2.85	21	3.16	149
B	16	2.39	21	3.16	197
C	19	2.85	21	3.16	144
D	18	2.7	21	3.16	179
E	15	2.24	21	3.16	168
F	15	2.24	21	3.16	129
G	20	3	21	3.16	182
H	17	2.54	21	3.16	159
I	3	0.39	21	3.16	63
J	12	1.77	21	3.16	92
K	17	2.54	21	3.16	155
L	13	1.93	21	3.16	93
M	21	3.16	21	3.16	234
N	17	2.54	21	3.16	177
O	21	3.16	21	3.16	173
P	16	2.39	21	3.16	152
Q	21	3.16	22	3.31	190
R	18	2.7	21	3.16	190
S	16	2.39	21	3.16	153
T	17	2.54	21	3.16	105
U	17	2.54	21	3.16	145
V	19	2.85	21	3.16	129
W	29	4.39	21	3.16	244
X	19	2.85	21	3.16	138
Y	19	2.85	21	3.16	102
Z	17	2.54	21	3.16	152

The height and width of each letter is specified as dot count and as degrees of visual angle. The latter was measured from the outer edges of the most extreme dots. Mean width of letters was 17.3 dots (2.60 arc°). Mean number of dots in the letter patterns was 153.6.

Each judged the displays from a distance of 3.5 m using both eyes, allowing correction of vision as needed. In each experiment the letters were displayed one at a time and respondents were asked to name the latter that was shown. They were required to make a guess even if they were unsure of which letter had been presented, and were not provided with feedback as to whether their answer was correct. For Experiment 2 used multiple flashes and respondents were also asked also to report whether or not they saw the display as flickering or as steady. Experiment 4 displayed each letter both with steady light emission and with multiple flashes, and respondents were asked also to report whether the two display conditions appeared to be equal in brightness.

Respondents generally named each letter without hesitation and the experimenter recorded the response. The time required for display and response was about 3–5 seconds per letter and the entire test session for a given experiment was completed in about 40 minutes.

### Ambient Illumination, Stimulus Timing, and Intensity

Ambient illumination was 10 lux, as measured using a calibrated Tektronix J 1811 photometer. This moderately dim lighting was provided by fitting standard fluorescent fixtures with occluding panels that reduced the quantity of illumination without changing the color temperature.

The letters were displayed on a 64x64 array of LEDs designated as the display board. Details on the design and operation of the display board are provided in Supplemental Methods of Greene & Ogden [5].

Letters were displayed in three different ways: a) a single and simultaneous flash of all the dots forming the letter pattern; b) multiple flashes of all the dots at specified frequencies for 750 ms; c) continuous emission from all the dots forming the letter pattern for 750 ms. For a given flash display voltage was applied to LEDs forming the letter pattern for one microsecond (μs). This duration is near the control limit of the display hardware and measures of light emission found that pulses approaching this limit manifest an “Off” spike that peaks with a constant intensity irrespective of the experimental intensity that was specified, and has a width at half height of 300 nanoseconds. This provides for a total pulse duration of 1.3 μs. The total energy of each flash corresponds to the area of the recorded trace, which is equal to duration multiplied by flash intensity. The intensities being reported were derived from the measures of pulse area, i.e., assuming the 1.3 μs duration and then calculating the intensity that would yield the total area of the trace.

Each LED in the array has a diameter that is less than five minutes of visual angle. Given this small size, it is more appropriate to consider the emission as coming from a point source than from a luminous surface, so emission has been specified in units of radiant intensity.

The measures of radiant intensity for steady emission were taken with a Thorlabs PM100USB radiometer with a S120C calibrated sensor. To assess the timing and intensity of the flashes, oscilloscope traces from a fast photodiode were captured. An Advanced Photonix PDB-C156 PIN silicon photodiode (15 ns rise time) was used in unbiased, unamplified photovoltaic mode, with an appropriate load resistor to convert the current output into a voltage which was measured by a 1X voltage probe. The voltage indicated on the oscilloscope trace was noted for steady emission and verified to be consistent with the signal recorded for brief flashes.

The preferred method for controlling LED intensity is with current regulation. However, it is not feasible to provide for 4096 individual control circuits, nor provide a single full-display current regulator, that could be controlled on a microsecond scale, with compensation for the rapidly varying load as the number of enabled LEDs are switched. Instead, a single programmable regulated voltage source was used, with a non-linear calibration table mapping voltage to measured intensity output.

### Experiments and Task Protocols

Extensive pilot work was done to determine the intensity ranges that would yield reliable data across a sample of randomly selected respondents. None of the individuals that were tested to establish effective ranges are included in the reported results. Once the treatment conditions to be applied in a given experiment were determined, every individual who was recruited for testing completed the task and no data was rejected as anomalous.

As described above, intensity of emission from the LEDs was based on the amount of voltage applied. Once the intensity range to be used was established, treatment levels were set at equal increments of the voltage across this range.

The order in which treatments were administered was random in each of the experiments described below. All 26 letters of the alphabet were displayed in a random order for each treatment level in each of the experiments.

Experiment 1 presented the letter patterns as a single brief flash of light, varying radiant intensity of the flash at the following 15 levels: 31.3, 40.5, 53.8, 70.7, 92.5, 118, 143, 173, 206, 244, 282, 329, 376, 423, 470 μW/sr. Correct naming of the displayed letter was scored as a one (1) and an incorrectly identified letter was scored as zero (0), with the proportion of correctly identified letters at a given intensity being designated as the “hit rate.”

Experiment 2 displayed each letter pattern as a sequence of brief flashes, varying the frequency at which flashes were delivered and with the full duration of the flash sequence being 750 ms. Each flash was delivered with a radiant intensity of 288 μW/sr, this being the intensity at which each respondent in Experiment 1 first reached or exceeded the 0.9 hit rate. Frequency was varied from 6–30 Hz in increments of 2 Hz, providing 13 treatment levels.

Experiment 3 displayed each letter with a sequence of brief flashes at 24 Hz, with the duration of the sequence being 750 ms. Flash intensity was varied to derive a multiple-flash activation curve, *i*.*e*., hit rate as a function of intensity when displayed with a 24 Hz sequence of flashes. Radiant intensity was delivered at 13 levels, these being: 15.38,. 15.39, 15.41, 15.45, 15.57, 15.84, 16.56, 18.35, 22.0, 29.0, 40.5, 57.2, 79.7 μW/sr.

Experiment 4 displayed each letter twice, once as a 24 Hz flash sequence and also with steady light emission; the order of the two conditions was random. The flash sequence and steady emission each lasted 750 ms and were separated by a 250 ms gap. Flash intensities were fixed at 288 μW/sr and the intensity of the steady display was varied across 13 levels, specified here in nanowatts per steradians: 7, 8, 9, 10, 12, 14, 16, 19, 21, 25, 31, 37, 43 nW/sr.

Raw data and data plots can be found in a supporting information file titled S1 Exp 1–4 Data. The data for each experiment is provided on a separate page of the spreadsheet file. Note that flash intensity levels for Experiments 1 and 3 are specified using the machine code (DN) that controlled the amount of voltage to be applied to the LEDs. Respondent judgments are coded as 1 or 0, representing that the letter was identified or not identified. The order in which the treatments were administered as well as the letter used for a given treatment was random. Therefore entries within a row of data reflect successive judgments for that treatment, but the letter presented on a given trial is not specified.

### Statistical Modeling

For each of the experiments random effects semi-parametric logistic regression was used to model the treatment effects. Conceptually, a smoothly varying “average” curve is fitted for the probability of correct response over all respondents, while the idiosyncratic deviations from the average response curve are incorporated using smooth person-specific random effects. This approach invokes only a minimal number of assumptions about the response curve—the main assumption is the lack of sudden jumps—and leads to fitted curves that follow the data closely. The downsides are that the parameter estimates are not meaningful, and the precision of the estimates is reduced compared to a well-fitting parametric curve. Technically, the average effect was modeled by a cubic spline with two to four equally spaced internal knots, while the person-specific deviations were modeled with a random intercept and a penalized cubic spline which had three equally spaced knots. For Experiment 2 the person specific penalized cubic spline was not significant, and was removed from the model.

In Experiment 2 the flicker-fusion threshold (50% median response) and confidence interval were found by numerical search. The median response occurs at 14.18 Hz, with the bounds of the 95% confidence interval being at 13.26 and 15.08 Hz. The confidence interval represents the interval of the average response, not the interval where 95% of people are expected to have the median response.

Analyses were performed using SAS version 9.3 (The SAS Institute, Cary, NC), using the Glimmix procedure for the primary analysis.

## Supporting Information

S1 Dataset This Excel file has four sheets, each containing the raw data of the eight subjects that were tested in Exps. 1–4. The treatment conditions for a given respondent are shown on successive rows, and the cell entries within the row indicates whether the letter was identified (1) or not (0) on the successive trials wherein that treatment was used.
(XLSX)
